# A convenient, optimized pipeline for isolation, fluorescence microscopy and molecular analysis of live single cells

**DOI:** 10.1186/1480-9222-16-9

**Published:** 2014-05-08

**Authors:** Jordan R Yaron, Colleen P Ziegler, Thai H Tran, Honor L Glenn, Deirdre R Meldrum

**Affiliations:** 1 Center for Biosignatures Discovery Automation, The Biodesign Institute, Arizona State University, Tempe, AZ, USA; 2 School of Biological and Health Systems Engineering, Arizona State University, Tempe, AZ, USA

**Keywords:** Single cell, RT-qPCR, Gene expression analysis, Fluorescence microscopy

## Abstract

**Background:**

Heterogeneity within cell populations is relevant to the onset and progression of disease, as well as development and maintenance of homeostasis. Analysis and understanding of the roles of heterogeneity in biological systems require methods and technologies that are capable of single cell resolution. Single cell gene expression analysis by RT-qPCR is an established technique for identifying transcriptomic heterogeneity in cellular populations, but it generally requires specialized equipment or tedious manipulations for cell isolation.

**Results:**

We describe the optimization of a simple, inexpensive and rapid pipeline which includes isolation and culture of live single cells as well as fluorescence microscopy and gene expression analysis of the same single cells by RT-qPCR. We characterize the efficiency of single cell isolation and demonstrate our method by identifying single GFP-expressing cells from a mixed population of GFP-positive and negative cells by correlating fluorescence microscopy and RT-qPCR.

**Conclusions:**

Single cell gene expression analysis by RT-qPCR is a convenient means for investigating cellular heterogeneity, but is most useful when correlating observations with additional measurements. We demonstrate a convenient and simple pipeline for multiplexing single cell RT-qPCR with fluorescence microscopy which is adaptable to other molecular analyses.

## Background

It is known that cellular heterogeneity is present even in seemingly homogenous, isogenic populations. This heterogeneity is observed in cell size, function and growth stage, and at both protein and gene transcript levels [1-3]. Despite the potential impact of investigating this heterogeneity, most of our understanding of disease pathology has been informed by bulk measurements made on cellular populations [4]. This approach is not optimal because population-averaged measurements are not always representative of the actual biological state or response. For example, multimodal responses become obscured and the contributions of rare, but important cells can be diluted beyond detection. Therefore, for many biologically and medically relevant questions, single cell resolution techniques are required [5-7].

Our lab and others have shown that performing gene expression analyses at the single cell level reveals useful information about disease states and conditional responses of both mammalian and bacterial cells [8-11]. However, these approaches rely on expensive, specialized equipment for automated cell sorting, or complicated and methodologically difficult manipulation tools. As a result, single cell gene expression experiments are often inaccessible to research labs with limited resources or expertise [11,12]. An additional limitation of existing methods is that chemical dissociation of samples is usually used to harvest cells for end-point analysis. This treatment has the potential to introduce physiological perturbations that may be reflected in variations in RNA species of interest. Further, during dissociation from an adherent population and processing by methods such as microcapillary aspiration or flow sorting, individual cells cannot be easily tracked. As a result, analyses done on live, adherent cells cannot be directly correlated with subsequent gene expression data for individual cells. Finally, custom-developed instrumentation, while enabling an individual lab to perform single cell experiments, may not be reproducible in other venues due to differences in protocols and sample handling. A comparison of the available methods for single cell isolation is given in Table 1. To address these challenges, we have optimized an adaptable pipeline for performing correlated live cell imaging and single cell reverse transcription quantitative polymerase chain reaction (RT-qPCR) which requires only broadly available equipment, minimal investment in consumables and minimal cell perturbation. We characterized our presented method for optimal single cell isolation and demonstrate its application by identification of GFP-expressing cells from among a mixed population with non-expressing cells both microscopically and by molecular detection using RT-qPCR on the same single cells.

**Table 1:** Comparison of current methods for single cell isolation

**Method**	**Advantages**	**Disadvantages**
Fluorescence-activated cell sorting	High throughput	High cost
	Single cell resolution	Specialized technical expertise needed
	Fluorescence-compatible	Suspended cells only
	Specific cell isolation	No cell-cell interaction capability
	Live cell compatible	Variable performance
Laser capture microdissection	Single cell resolution	Low throughput
	Fluorescence-compatible	High cost
	Specific cell isolation	
	Live cell compatible	
Laser capture microdissection	Specific cell isolation	Specialized technical expertise needed
	Compatible with tissue samples	Infrequently compatible with live cells
	Capable of cell-cell interaction studies	Potential neighbouring cell contamination
		Need to identify cell of interest
		Adhered cells only
		Variable performance
Microcapillary aspiration	Single cell resolution	Low throughput
	Fluorescence-compatible	High cost
	Live cell compatible	Necessary technical expertise
	Capable of cell-cell interaction studies	Suspended cells only
		Variable performance
Microfluidics	Variable throughput	Specialized technical expertise needed
	Variable cost	Generally specialized per experiment
	Single cell resolution	Random cell isolation
	Fluorescence-compatible	Variable performance
	Live cell compatible	
	Adherent or suspended cells	
	Capable of cell-cell interaction studies	
Terasaki plate and dilution	Low cost	Mid to low throughput
	Low technical complexity	Random cell selection
	Single cell resolution	
	Fluorescence-compatible	
	Live cell compatible	
	Adherent or suspended cells	
	Capable of cell-cell interaction studies	
	Consistent performance	

Terasaki-style microtest assays were developed in the 1960s by Paul Terasaki for tissue-typing microcytotoxicity tests on human leukocyte antigens with only one microliter of patient antiserum [13]. Modern Terasaki plates are generally made of optically clear polystyrene with flat-bottomed wells accommodating approximately 20 μL volumes each. While still used for their original intended microcytotoxicity purposes, Terasaki plates have also been used for isolation cloning, because, after plasma treatment to promote cell adhesion, they provide a small, fluid-isolated culture environment for growth [14]. Because of the small volumes, ability to support adherent cell culture and compatibility with microscopic observation, Terasaki plates are excellent candidate substrates for designing a single cell RT-qPCR assay. These commonly available substrates are underutilized in the literature for single cell RT-qPCR analysis and have only been demonstrated for single-plex identification of gene expression [15]. Further, the previously published, and rarely reported, application of this substrate for single cell RT-qPCR is non-optimized and only briefly described thereby requiring substantial preliminary work for groups wanting to use this technique.

**Figure 1:**
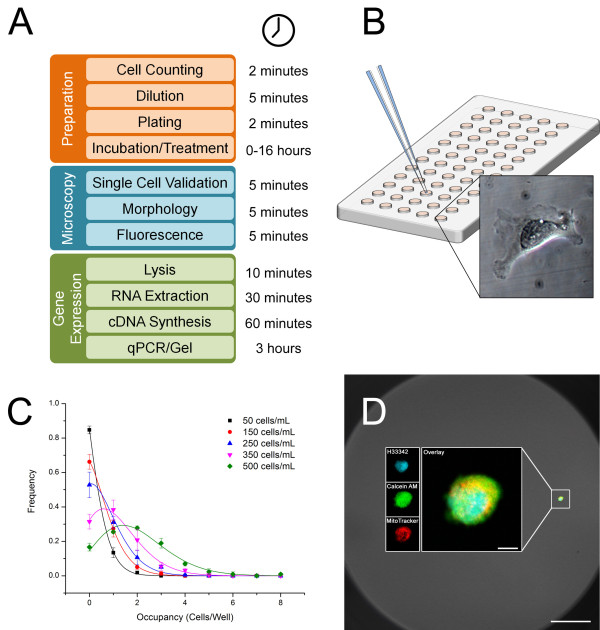
**Schematic overview of the pipeline. A)** Succinct overview of the pipeline, sectioned into three main processes: preparation, microscopy, and gene expression. Approximate time per plate for each step in the procedure is shown. **B)** Diagram of the cell isolation process. Diluted solutions of cells are dispensed into fluidically isolated wells of a Terasaki plate. Inset illustrates the spreading morphology of a single adherent cell on the plate. **C)** Concentration curve experiments with MDA-MB-231 cells demonstrating the ability to tune the well occupancy by altering initial seeding concentration according to Poisson statistics. Approximately 200–300 cells/mL was identified as the optimal concentration for obtaining single cells. Error bars represent standard deviation and curves represent Poisson fit. **D)** Demonstration of three-color fluorescence on the Terasaki plates. An isolated THP-1 cell is stained with Hoechst 33342 (DNA; blue), Calcein AM (cell membrane integrity; green) and MitoTracker CMXRos (mitochondria; red). Main scale bar represents 100 μm and inset scale bar represents 5 μm.

Here, we describe the optimized application of Terasaki plates for single cell RT-qPCR, an expansion of the pipeline to include correlated molecular analysis with fluorescence microscopy, and a step-wise protocol with troubleshooting guidelines. Major advantages of the method described here versus existing methods include low method adoption cost and learning curve, broad compatibility with various detection chemistries and microscopic methods, and multiplexing analysis of visual observations and molecular detection in the same single cells. The presented pipeline was designed by combining and characterizing simple, inexpensive and reliable methods to reduce costs and maximize broad applicability (Figure 1A-B). Briefly, we isolate single cells by the following steps: 1) establish cell density using a cell counter, 2) determine the optimal cell density required to achieve one single cell per well in a Terasaki plate, 3) homogenize the suspension and dispense 10 μL into each well using a standard hand-held micropipette, 4) incubate cells for approximately 10–20 minutes in either a tissue culture hood or a 37ºC incubator, 5) verify and score positive single cells in each well. As demonstrated, the resulting single cells can be used for a number of downstream applications including experimental treatments, fluorescence microscopy and RT-qPCR analysis.

## Results

### Preparation of Terasaki plates for improved cell attachment and viability

We first sought to determine if single cells could survive overnight incubation under fluidic isolation and limited medium in Terasaki-style microtest plates. We hypothesized that overnight viability (as determined by spreading morphology) could be improved by plasma treating to decrease hydrophobicity compared to untreated controls [16]. Our qualitative observations suggest that a brief, 1 minute plasma treatment under a 500 mTorr vacuum and 10.15 W RF-power was sufficient to improve spreading for weakly or moderately adherent cell lines while strongly adherent cell lines and suspension cell lines did not show a noticeable difference due to plasma treating. Coating the wells with attachment-enhancing polymers such as PEI, poly-d-lysine or collagen may be sufficient alternatives to plasma treatment in the absence of available equipment, but will require additional characterization and optimization for the cell types and biological conditions being studied [17]. In addition to spreading morphology and division during extended incubation, cells retained calcein AM, an indicator of plasma membrane permeability and general cell viability (Figure 1D).

### Single cell isolation by stochastic seeding

To achieve a suitable occupancy frequency of one cell per well, we identified optimal cell concentration by plating different cell densities into Terasaki plates using a principle similar to limiting dilution cloning [18]. We explored concentrations of cells ranging from 50 cells/mL to 500 cells/mL and seeded them according to our protocol. Once settled, each plate was counted for complete well occupancy and distributions were determined (Figure 1C). The distributions determined from these experiments were verified with at least three adherent cell lines and a suspension line and consistently resulted in 15–25 wells per plate with live single cells. The resultant well occupancy followed distributions as expected by Poisson statistics with high reliability (R^2^ > 0.98). There was no appreciable difference between 200 and 300 cells/mL, though lower densities resulted in fewer multi-cell wells. Accordingly, 200–300 cells/mL was found to be the optimal condition for maximizing single cell well occupancy. Adherent and suspension cells exhibited very similar seeding statistics. Seeded cells could be subsequently stained with vital dyes or specific probes and imaged on multiple fluorescent channels with sufficient resolution to detect subcellular distributions (Figure 1D).

### Correlated observations of fluorescence and gene expression in the same single cells

**Figure 2:**
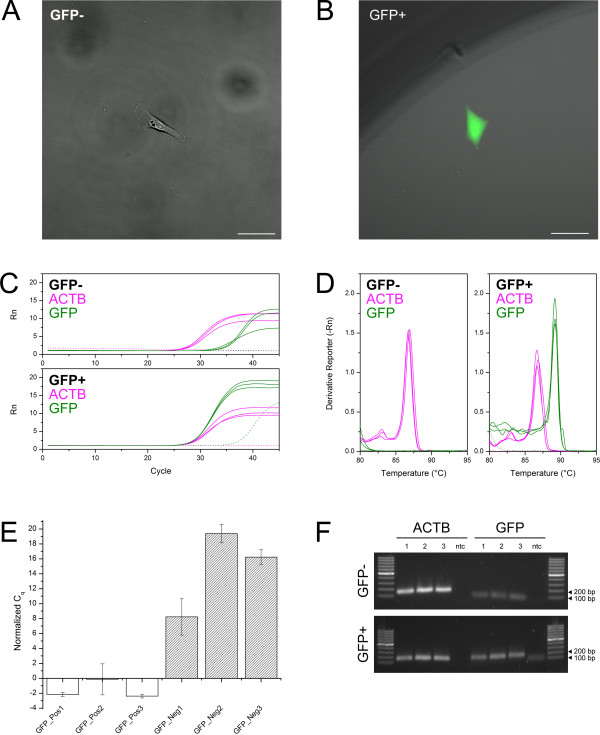
**Identification of single GFP-positive and negative cells from a mixed population. A-B)** Adherent GFP-negative and GFP-positive cells obtained by the described cell isolation method and observed by fluorescence microscopy. **C)** qPCR curves demonstrating the ability to differentiate between GFP-negative (top) and GFP-positive (bottom) cells without pre-amplification. Two gene targets were identified in each single cell: beta-actin (magenta) and GFP (green). The delayed amplification shown in the GFP-negative curves are caused by primer dimers, as supported by melt curve analysis, agarose gel electrophoresis and DNA sequencing. **D)** Melt curve analysis showing the identification of individual peaks corresponding to the presence or absence of GFP (green), while beta-actin is observed at similar levels in both samples (magenta). **E)** Analyzed data for three GFP-positive (left group) and three GFP-negative (right group) cells isolated from a mixed population of cells. Results for each single cell were normalized to expression of beta-actin (ACTB) and reported as normalized C_q_, which is defined as C_q, GFP_ - C_q, ACTB_. Error bars represent standard deviation of 3 technical replicates of divided samples from individual cells. The difference between normalized C_q_ from GFP+/- is significant as determined by *T*-test with *p* < 0.05. **F)** Validation gel illustrating the presence of beta-actin in both cells, but a differential presence of GFP amplification in cells which were observed to be GFP-positive versus GFP-negative. Off-target bands in the negative control are primer dimers as confirmed by melt-curve analysis.

To demonstrate the ability of the pipeline to identify specific signatures of single cells, we measured the presence of GFP transcripts in isolated cells from a population containing a mixture of GFP-positive and GFP-negative cells (Figure 2). We sought to determine whether the volumes attainable in the Terasaki plates would allow detection of GFP transcripts from GFP-positive cells that could be correlated with fluorescence observations from the same sample. The GFP-positive cells used in these experiments were CP-D cells (ATCC® CRL-4030™), an hTERT-immortalized cell line representing high-grade dysplasia in Barrett’s esophagus that was stably transfected with a plasmid containing the GFP coding sequence. The GFP-negative cells were CP-A cells (ATCC® CRL-4027™) a related hTERT-immortalized cell line representing non-dysplastic metaplasia in Barrett’s esophagus. Both of these cell lines were mixed 1:1 prior to being seeded on a Terasaki plate for single cell isolation. Single cells were scored and GFP-positive and –negative cells were identified by fluorescence microscopy and subsequently isolated for gene expression analysis (Figure 2A-B). Total RNA was purified from each collected single cell and the entire collected eluate was used in independent reverse transcription reactions to produce cDNA. Subsequently, the cDNA was divided into three replicates for the target gene, GFP, and three replicates for the control gene, beta-actin. Simultaneous no template controls were run in parallel. Reproducibility of this method was good, as representatively indicated by the tight distribution of the amplification curves in Figure 2C and the height of the peaks in the melt curves in Figure 2D. As is commonly observed in RT-qPCR using intercalating chemistries (e.g., SYBR), occasional primer dimer amplification occurred, as seen in the late-rising dotted green amplification curve in the lower panel of Figure 2C. Primer dimer amplification is identified and distinguished from sample amplification by the characteristically late C_q_ value, lack of expected melt curve peak and small band size (Figure 2C-D and Figure 2F).

A challenge in single cell analysis is the ability to discriminate between variability due to error in a method and real differences due to biological heterogeneity and gene expression stochasticity. Using the presented pipeline, the data collected by RT-qPCR and melt curve analyses illustrated marked differences in GFP mRNA levels between isolated cells from a mixed population that corresponded to positive and negative fluorescence observations (Figure 2C-D). Normalized C_q_ analysis (C_q,GFP_ - C_q,ACTB_) demonstrated a significant difference in signal between GFP-positive and negative cells as determined by *T*-test with *p* < 0.05 (Figure 2E) [19]. A gel electrophoresis analysis was performed to validate the qPCR data according to expected amplicon sizes, which are described in the Methods section (Figure 2F). The results were further confirmed by band extraction and DNA sequencing, resulting in nucleotide sequences corresponding to the two expected gene targets. These results show that the volumes attainable in the Terasaki plate yield sufficient sample concentration to quantify gene expression of single cells for the purpose of population discrimination despite the inherent difficult in identifying variability due to error or endogenous heterogeneity and stochasticity.

## Discussion

While single cell studies have the potential to reveal important heterogeneity in a wide variety of biological systems, the ability to perform the techniques required for single cell analysis are commonly limited by a laboratory’s technical expertise and available instrumentation. We sought to develop a simple protocol for performing single cell gene expression studies which is accessible to any lab already performing similar studies on bulk samples.

We have demonstrated a simple and effective method for isolating live single cells for microscopic imaging and gene expression analysis by RT-qPCR. The major advantages of our method over previous approaches include: 1) the use of commonly available consumables circumventing the need for expensive equipment, 2) improved throughput of single cell selection compared to other manual methods due to random seeding and direct verification of well occupancy and viability, 3) a simplified single cell isolation procedure with minimal physical and chemical manipulation of cells, 4) total RNA extraction compatible with detection of multiple gene targets, and 5) multiplexed single cell imaging and gene expression analysis. Further, our method is compatible with a wide range of chemistries, allowing integration into experimental protocols that include various drug treatments or fluorescent indicators. All steps can be carried out under standard aseptic cell culture conditions and cell viability is not compromised. Suggested improvements to the presented protocol such as electronic repeating pipettes or fluid handling robots may require additional purchases, but will improve throughput; we were able to reduce the time to seed one plate from approximately 5 minutes to less than 45 seconds with an electronic repeating pipette. Also, RNA isolation and purification may be avoided by using one-step RT-qPCR reagents, though this comes at the cost of reducing the number of gene targets per single cell sample. Additionally, the use of Taqman or other hydrolysis probe chemistries can improve the amplification specificity, but may result in considerably more expensive up-front costs per reaction.

The ability to multiplex visual observations of cells with molecular analysis is essential to understanding dynamic responses of cells to external perturbation. The method reported here provides a straightforward and effective procedure for achieving multiplexed visual and molecular analysis at the single cell level. We anticipate that this method will be extensible to the analysis other biomolecules (e.g., proteins) at the single cell level using assays such as proximity ligation assay-qPCR [6]. Further, the use of live-cell fluorescent reporters can facilitate the tracking of intracellular events for improved temporal correlation with molecular analysis. For example, the nuclear translocation of a fluorescently tagged transcription factor can be tracked and then correlated to the production of mRNA transcripts that are regulated by that transcription factor.

## Conclusions

This simple and flexible method for single cell analysis will lower the barrier to entry in this field of study and accelerate the identification of heterogeneity in populations and the discovery of important biosignatures of disease.

## Methods

Brief descriptions of methods specific to this study are provided below followed by a detailed, step-wise protocol.

### Cell culture

CP-A (ATCC® CRL-4027™) and TurboGFP-expressing CP-D cells (ATCC® CRL-4030™; transduced with MISSION® pLKO.1-puro-UbC-TurboGFP™) were maintained in serum-free Keratinocyte medium modified with 20 ng/mL epidermal growth factor, 140 μg/mL bovine pituitary extract, 100 U/mL penicillin and 100 μg/mL streptomycin (Gibco). Cells were maintained at 37°C under 5% CO_2_ in a humidified atmosphere. Cells were trypsinized with 0.05% Trypsin-EDTA for 10 minutes, centrifuged at 900 rpm for 3 minutes and counted using the Trypan Blue assay on a Countess® automated cell counter (Life Technologies); only passages identified as greater than or equal to 95% viable were utilized in experiments. Cells were resuspended at 200–300 cells/mL or in a 1:1 mixture unless otherwise noted. THP-1 (ATCC® TIB-202™) cells were cultured per ATCC instructions and used for determining well occupancy in preliminary concentration curve experiments as well as the three-color fluorescence data. MDA-MB-231 (ATCC® HTB-26™) cells were cultured at 37°C under 5% CO_2_ in a humidified atmosphere in complete DMEM supplemented with 10% FBS, 100U/mL penicillin and 100 μg/mL streptomycin (Gibco) and subcultured as described for the CP-A and CP-D cells. MDA-MB-231 were used for determining well occupancy in preliminary concentration curve experiments.

### Preparation of Terasaki plates

Terasaki-style microtest plates (#470378, Thermo Scientific, Pittsburgh, PA) were briefly cleaned using pressurized nitrogen gas to remove particulate from the well area. The plates were then exposed to air plasma in a plasma cleaner (PDC-001, Harrick Plasma, Ithaca, NY) for 1 minute under 500 mTorr vacuum with 10.15 W RF-power; we noticed a decrease in the time necessary for cell spreading after plasma treating, but this step is not required. The outer surfaces of the plates were sprayed with 70% ethanol and allowed to dry in a sterile, laminar flow hood prior to cell seeding.

### Calibration curve of cell isolation

Cells were suspended at a concentration of 50–500 cells/mL in increments of 50 cells and seeded onto Terasaki plates as described in the step-wise protocol. Whole plates were manually counted and scored for cell occupancy per well.

### Phase contrast and fluorescence microscopy

Plates were briefly observed by phase contrast microscopy on a Nikon TS-100 microscope with 10 × and 20 × objectives and scored for viability as “live” or “dead” based on spreading morphology and phase contrast characteristics. Wells identified as containing a live single cell were further observed for fluorescence on an EVOS® FLoid® Cell Imaging Station (Life Technologies, Carlsbad, CA) using the white and green light detection options. For testing three-color fluorescence compatibility, live THP-1 cells were loaded with 10 μg/mL Hoechst 33342, 500 nM MitoTracker CMXRos and 2 μM Calcein AM (Life Technologies) and imaged on a Nikon TE2000 inverted microscope with a C2 confocal scanner (Nikon Instruments, Melville, NY).

### Total RNA extraction and reverse transcription

Samples were harvested from individual wells containing single cells, kept at -80°C until further use (less than one week) and subsequently processed for RNA extraction and purification as described in the step-wise protocol. Total RNA was eluted to a final volume of 9 μl. First-strand cDNA synthesis was performed in a thermal cycler (Life Technologies) with conditions as described in the step-wise protocol. cDNA was stored at -20°C until further use.

### qPCR and validation

qPCR was performed as detailed in the step-wise protocol. Primers used are described in Table 2. Three technical replicates and a no-template control reaction were performed for each gene in each sample. A StepOnePlus™ Real-Time PCR System (Life Technologies) was used for thermal cycling as described in the step-wise protocol. Data was analyzed using StepOne™ Software version 2.1 (Life Technologies). Results were confirmed via 1% agarose gel electrophoresis and melting curve analysis. Primers were validated by band extraction from the agarose gel (QIAquick Gel Extraction kit, Qiagen, Germantown, MD) followed by sequencing.

**Table 2:** RT-qPCR primers

**Gene target**	**Accession #**	**Forward sequence**	**Reverse sequence**	**Amplicon size**
Beta-actin (Human)	NM_001101.3	5′-ctggaacggtgaaggtgaca	5′-aagggacttcctgtaacaacgca	140 bp
GFP (TurboGFP)	GU452685.1	5′-aggacagcgtgatcttcacc	5′-cttgaagtgcatgtggctgt	164 bp

### Data analysis and figures

Statistical *T*-test analysis was performed in OriginPro 8.1 (OriginLab, Northampton, MA) and Excel 2010 (Microsoft, Redmond, WA). Results were considered significant at *p* < 0.05 and plotted error bars represent standard deviation of normalize C_q_ among triplicate technical replicates. Images were analyzed in NIS-Elements AR 3.21 (Nikon Instruments). Figures were produced in OriginPro 8.1, Photoshop CS3 Extended (Adobe, San Jose, CA) and PowerPoint 2010 (Microsoft).

### Step-wise protocol

#### Reagents and equipment

• Countess® automated cell counter (Life Technologies, Carlsbad, CA, #10227)

• Countess® Cell Counting Chamber Slide (Life Technologies, #C10228)

• Trypan Blue Stain, 0.04% (Life Technologies, #T10282)

• 72-well Terasaki plates (Fisher Scientific, Pittsburgh, PA, #470378)

• Quick-RNA™ MicroPrep kit (Zymo Research, Irvine, CA, #R1051)

• SYBR Premix Ex Taq™ II (2X) (TaKaRa/Clontech, Mountain View, CA #RR820)

• PCR plates

o e.g., TempPlate No-skirt 0.1 mL PCR plates (USA Scientific, Ocala, FL, #1402-9590)

• Optically-clear PCR film

o e.g., TempPlate RT optically clear film (USA Scientific, Ocala, FL, #2978-2100)

• Gel imaging equipment

o e.g., Bio-Rad Gel Doc (Bio-Rad, Hercules, CA, #170-8170)

• Thermal cycler

o e.g., Veriti® 96-well Thermal Cycler (Life Technologies, #4375786)

• qPCR instrument

o e.g., StepOnePlus™ Real-Time PCR system (Life Technologies, #4376598)

• General lab supplies (microcentrifuge tubes, pipettes, etc.)

• Inverted microscope

o Minimum brightfield (preferred with phase). Optionally with fluorescence capabilities appropriate for the fluorophores applied.

#### Preparation

##### Cell counting

Note: This section assumes the availability of a Countess® automated cell counter. Adjust protocol as needed for available cell counting method.

1. Culture cells as appropriate and prepare for counting as necessary (e.g., trypsinization or aspiration).

2. Transfer 10 μL of cell suspension to a 1.5 mL microcentrifuge tube.

3. Add 10 μL of Trypan Blue stain and pipette up and down to mix.

4. Transfer 10 μL of mixed solution from step 3 to a Countess® Cell Counting Chamber Slide.

5. Insert Countess® Cell Counting Chamber Slide into Countess® instrument, focus image and run program.

##### Dilution

1. Calculate appropriate dilution process to obtain a solution with 200–300 cells/mL cell density. For example:

Stock #1: 5e5 cells in 1 mL (live cell count)

Dilution #1: Add 20 μL of Stock #1 to 980 μL medium (10,000 cells/mL)

Dilution #2: Add 20 μL Dilution #1 to 980 μL medium (200 cells/mL).

##### Plating

1. Briefly agitate the microcentrifuge tube to disperse the diluted cells prior to plating.

2. Dispense 10 μL of diluted cell solution (Dilution #2 in example above) into each well of a 72 well Terasaki plate with a manual or electronic repeating pipette.

##### Incubation and treatment

1. Transfer plate to an incubator or leave in a cell culture hood for a minimum of 10–20 minutes.

2. (Optional) Incubate cells overnight or as necessary for full adhesion.

3. (Optional) Expose cells to drugs or other treatments as desired.

4. (Optional) Treat cells with vital dyes or fluorescent indicators.

#### Microscopy

##### Well occupancy

1. Check cell viability and well occupancy with an inverted microscope at 10 × magnification, ideally with phase contrast.

2. On a 6×12 spreadsheet, mark the wells which contain live single cells.

##### Live cell analysis

1. Image wells with single live cells by fluorescence or bright-field microscopy to record morphology or assay physiological indicators.

#### Gene expression

##### Lysis

**Figure 3:**
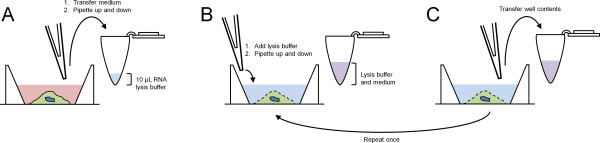
**Cell lysis procedure.** A visual reference for performing cell lysis as described in the step-wise protocol. **A)** Total medium from target wells are transferred to a PCR tube containing 10 μL RNA lysis buffer and pipetted up and down. **B)** RNA lysis buffer is added to the target well to the same PCR tube as in **A**. Procedures **B** and **C** are repeated once for a total of 3 transfers from each target well.

1. Prepare 1 PCR tube per target well by adding 10 μL RNA lysis buffer (from Zymo kit) or other appropriate lysis buffer.

2. Transfer complete volume from desired sample wells to individual PCR tubes containing RNA lysis buffer and pipette up and down to ensure no loss of material in pipette tip (Figure 3A).

3. Add 10 μL RNA lysis buffer to each well, pipette up and down, and transfer volume to the PCR tube corresponding to the target well. Repeat once for a total of three transfers from each target well. At the end of this step there should be 1 PCR tube containing target samples for each target well in 40 μL RNA lysis buffer (Figure 3B-C).

4. Use samples immediately, or if there are a large number of samples to harvest store at or below -80°C.

##### RNA extraction

Note: All components from Quick-RNA™ MicroPrep kit; other kits may be adequate but should be evaluated individually.

1. Transfer lysate to Zymo-Spin™ IC column in a 2 mL collection tube.

2. Centrifuge at 12,000× g for 1 minute. Discard flow-through.

3. Add 400 μL RNA Pre-Wash Buffer to column and centrifuge at 12,000× g for 1 minute. Discard flow-through.

4. Add 700 μL RNA Wash Buffer to column and centrifuge at 12,000× g for 1 minute. Discard flow-through. Repeat with 400 μL RNA Wash Buffer.

5. Centrifuge column and emptied collection tube at 12,000× g for 2 minutes.

6. Place column in an RNase/DNase free 1.5 mL microcentrifuge tube. Add 9 μL DEPC-treated water and let sit for 1 minute. Centrifuge at 12,000× g for 1 minute. Use purified total RNA immediately or store at or below -80°C.

##### cDNA synthesis

1. Prepare a master mix of qScript™ cDNA SuperMix (Quanta Biosciences #95048) based on the following components for 1X reaction:

qScript™ cDNA SuperMix      2 μL

DEPC H_2_O            1 μL

Total RNA            7 μL

2. Cap reactions, vortex and centrifuge briefly.

3. Perform first strand cDNA synthesis in a thermal cycler (Table 3).

4. Store cDNA at or below -20C or use immediately.

**Table 3:** cDNA synthesis conditions

**Cycle #**	**Temperature**	**Time**
1	25°C	5 minutes
2	42°C	30 minutes
3	85°C	5 minutes
4	4°C	Hold

##### qPCR

1. Prepare a master mix of SYBR Premix Ex Taq™ II (2X) based on the following components for a 1X reaction:

SYBR Premix Ex Taq™ II (2X)    5 μL

4 μM Forward Primer       0.4 μL

4 μM Reverse Primer       0.4 μL

ROX Reference Dye        0.2 μL

DEPC H_2_O            2.0 μL

2. Add 8 μL of master mix to each well of a 96-well PCR plate.

3. Add 10 μL DEPC H_2_O to each tube of the template, vortex briefly and centrifuge.

4. Add 2 μL of cDNA template or DEPC H_2_O (for no-template controls) to each corresponding well.

5. Place optically clear PCR plate film on the PCR plate and rub over the top with a lab wipe to seal each well.

6. Place plate in plate centrifuge and run for 1 minute at 1000 rpm.

7. Place tubes in StepOnePlus™ machine, or available qPCR instrument, and run program (Table 4).

8. Perform data analysis in StepOne™ Software version 2.1 or higher, or software compatible with available instrumentation.

**Table 4:** **qPCR conditions**^**1**^

**Cycle #**	**Temperature**	**Time**
1	95°C	30 seconds
2-42	95°C	5 seconds
	60°C (Collect Data)	30 seconds
43	Melt Curve Analysis	Instrument Dependent

^1^Thermal cycling conditions may vary depending on reagent manufacturer.

##### Confirmation gel

1. Prepare a 1-2% agarose gel (e.g., Lonza SeaKem LE) in 1X TAE or TBE buffer with available fluorescence dye (e.g., SYBR Safe).

2. Add loading dye to PCR-amplified sample and mix by pipetting up and down.

3. Add sample and 50 bp or 100 bp ladder to agarose gel and run at 90–120 V for 45 minutes.

4. Image gel.

5. Compare gel bands to qPCR curves to validate quality of isolation and gene amplification.

### Troubleshooting

Troubleshooting advice can be found in Table 5.

**Table 5:** Troubleshooting

**Problem**	**Possible reason**	**Solution**
Cells are not viable after seeding onto Terasaki plates	Cells were not viable prior to seeding	Check viability using Trypan Blue dye exclusion or fluorescent stain system (e.g., Calcein AM/Ethidium Homodimer-1) to ensure cells are viable prior to seeding
	Terasaki plates are contaminated	Confirm sterility of plates and treat as necessary by UV sterilization and/or alcohol soaking.
Too many/too few cells per well	Inaccurate cell dilution	Confirm cell counting method accuracy and recalibrate any automated instrumentation
	Cells are adhering to the sidewalls of the wells	First confirm cells are deposited into the well by visually identifying sidewall-adhered cells. Next, allow cells to settle without disruption after seeding or, alternatively, centrifuge plates for 1minute at 900 rpm to draw cells to the bottom of the well.
No amplification of target gene	Improperly designed primers	Confirm primer design by bulk cell RT-qPCR followed by band sequencing and redesign primers as necessary.
	Loss of RNA during harvesting	Reduce number of fluid transfer steps and/or use low-binding tubes and tips.
	RNA degradation	Use RNase-free reagents and perform all steps following cell harvesting on ice.

## Abbreviations

RT-qPCR: Reverse transcription-quantitative polymerase chain reaction; GFP: Green fluorescence protein.

## Competing interests

The authors declare that they have no competing interests.

## Authors’ contributions

JRY and DRM conceived of the study. JRY and CPZ developed protocols and collected all data. JRY, CPZ and THT analysed the data. THT, HLG and DRM provided direction and advisement. JRY prepared the manuscript and all authors edited the manuscript. All authors read and approved the final manuscript.
